# Facteurs de risques de mortalité néonatale dans l'hôpital de gynécologie-obstétrique de la wilaya de Sidi Bel Abbes, Algérie

**DOI:** 10.11604/pamj.2015.20.387.5032

**Published:** 2015-04-20

**Authors:** Harir Noria, Ourrad Sarah, Ourrad Asmaa

**Affiliations:** 1 Laboratoire de Microbiologie Moléculaire, Proteomics et Santé, Département de Biologie, Université Djillali Liabès de Sidi Bel Abbes (UDL-SBA), Algérie

**Keywords:** Mortalité néonatale, fréquence, facteurs de risques, wilaya de Sidi Bel Abbés, neonatal mortality, frequency, risk factor, wilaya of Sidi Bel Abbés

## Abstract

**Introduction:**

Il s'est agit ici de déterminer la fréquence et les facteurs de risques de mortalité néonatale au service néonatologie de l'Etablissement Hospitalier Spécialisé Gynécologie Obstétrique de la wilaya de Sidi Bel Abbés (Ouest Algérien).

**Méthodes:**

Il s'agit d'une étude rétrospective a visée descriptive et analytique porté sur tous les décès de 2011-2012 survenus au service de néonatologie de Sidi Bel Abbes.

**Résultats:**

Au total 1209 cas de mortalité néonatale ont été enregistré durant les deux années (2011-2012), soit une fréquence de 5.3%. Il s'agissait dans 96,85% des cas de mortalité précoce. La mortalité néonatale étant multifactorielle, l'analyse statistique a pu incriminer de façon majoritaire: l’âge maternel avancé (>35) (OR = 3.1; IC 95% (2.30 -4.40); p = 0.001); la multiparité (OR = 8.15; IC 95% (2.85-10.05); p = 0.001); l'infection génitale(OR = 5.3; IC 95% (2.5-6.7); p = 0.001); la prématurité (OR = 10.08; IC 95% (3.45-12.02); p = 0.001); le faible poids de naissance (OR = 4.5; IC 95% (1.6-10.5); p = 0.001); l'ictère (OR = 4.8; IC 95% (1.26-6.02; p = 0.001) et la souffrance fœtale aigue (OR = 3.4; IC 95% (0.89-5.14); p = 0.001).

**Conclusion:**

Une prise en charge efficace de la grossesse et du nouveau-né dans sa première semaine de vie, devraient amélioraient le pronostic néonatal.

## Introduction

La naissance d'un enfant mort-né ou une perte cruelle d'un nouveau-né constitue un drame familial auquel on tente de trouver des explications souvent fatalistes. A l'heure actuelle sur les 130 millions de naissances annuelle, on compte 4 millions de décès de nouveau-nés dont 75% meurent au cours de la première semaine de leur vie [1]. 98% des nouveaux nés concernés vivent dans les pays à faible revenus ou les taux de mortalités néonatales restent élevés de plus de 45% en moyennes contre 4% dans les pays développés [2]. Les causes et les facteurs de risque sont bien connus actuellement et accessibles à la prévention. Près de 70% des décès pourraient être évités par des interventions simples et à moindre coût avant et pendant la grossesse, durant l'accouchement et même pendant la période du post partum [3]. En Algérie, l'examen de l’évolution de la mortalité infantile entre l'année 1990 et l'année 2012, a montré que le niveau de mortalité infanto- juvénile s'est réduit d'un peu plus que la moitié; en passant de 55,7‰ à 26,1‰. De même le taux de mortinatalité s'est réduit de 21,4‰ à 15,9‰ durant cette même période [4]. Ainsi, la morbidité, la mortalité et les caractéristiques épidémiologiques infanto- juvénile ont connu de nettes modifications durant cette décennie. L’étude de ces paramètres nous permet de suivre leurs tendances évolutives au fil des années, de reconnaître les nouveaux besoins de la population et d'entreprendre des mesures adaptées. D'où la nécessité d'un recueil régulier des données épidémiologiques. C'est dans ce contexte que se situe cette étude rétrospective sur la mortalité néonatale révolue dans l'Etablissement Hospitalier Spécialisé Gynécologie Obstétrique (E.H.S.P.G) de la wilaya de Sidi Bel Abbés (Ouest Algérien).

## Méthodes

Il s'agit d'une étude rétrospective a visée descriptive et analytique porté sur tous les décès de 2011-2012 survenus en E.H.S.P.G de Sidi Bel Abbes et Hospitalisés au premier jour de vie dans le service de néonatologie. Notre étude a recensé 1209 nouveau-nés décédés entre 0 et 27 jours révolus au service de néonatologie. Nous avons essayé dans cette étude de recueillir le maximum de donnés à partir des dossiers Obstétricaux archivés et les fiches des nouveau-nés au service de néonatologie. Les critères d’évaluations ont été adaptés a fin de réduire les pertes d'informations, les variables retenus étaient: ***a)Données sociodémographiques:*** l’âge de parturientes, la parité des parturientes, le niveau socio-économique. ***b) Antécédents médicaux:*** mort fœtale in-utéro, avortement, grossesse extra utérine. ***c)Pathologies survenues au cours de la grossesse:*** diabète, Hypertension artérielle (HTA), anémie. ***d) Renseignements sur le nouveau-né:*** poids, sexe, score d'Agars, terme de la grossesse. ***e) Données étiologiques:***: les causes de décès, les facteurs de risques, délai de survenue des décès. La saisie des données a été faite sur ***l'Excel 2007*** puis le traitement statistique a été effectué grâce au logiciel ***Statview 1995***. Les variables associées à la mortalité à p<0,05 à l'analyse univariée étaient incluses dans la régression logistique pour déterminer les facteurs qui lui sont indépendants.

## Résultats

Sur les 22780 accouchements survenus à la maternité de la région de Sidi Bel Abbes entre 2011-2012, nous avons enregistré 1209 cas de mortalité néonatale(MNN), soit un taux global moyen de 5,3%. Le Tableau 1, illustre l'évolution de la mortalité néonatale au cours de la période 2011-2012 au niveau de la maternité de Sidi Bel Abbés.

**Tableau 1:** Evolution de la mortalité néonatale au cours de la période 2011-2012 à la Maternité de Sidi Bel Abbés

Année	2011	2012
Nbr de naissance	11320	11460
Nbr de MNN	621	588
Pourcentage%	51.36%	48.64%

### Caractéristiques descriptives maternelles

L’âge moyen des parturientes était de 34 ans. Les résultats statistiques ont révélé que la mortalité néonatale(MNN) était plus élevé dans la tranche d’âge plus de 35 ans avec une fréquence de 53.26% suivie de celle dans la tranche d’âge 20-35 soit a 28.53%. Le mode d'accouchement par césarienne était le plus pratiqué pour ces parturientes soit à 65.83% versus 29.36% par voie basse. Ces patientes étaient des multipares à 52.52% et durant leur grossesse, elles soufraient majoritairement d'infection génitale à 49.39% et d'hypertension artérielle à 26.82%. Quarante-sept cas présentaient des antécédents obstétricaux dominés par les avortements à 42.55%, suivie respectivement par enfants décédés (25.53%) et mort-nés (21.27%). La moitié de ces parturientes provenaient d'un milieu socio-économique modeste (Tableau 2).

**Tableau 2:** Description des caractéristiques maternelles des nouveau-nés décédés

Caractéristique maternelle	Nombre	Pourcentage%
**Age**		
< 20 ans	220	18.19
20-35 ans	345	28.53
>35 ans	644	53.26
**Parité**		
1	323	26.71
2-3	251	20.76
>3	635	52.52
**Pathologies associées à la grossesse**		
Diabète	15	9.14
HTA	44	26.82
Anémie	15	9.14
Myopie	1	4.87
Cardiopathie	8	6.09
Infection génitale	81	49.39
**Antécédent obstétricaux**		
ABRT	20	42.55
MIU	5	10.63
Mort-né	10	21.27
Enfant décédé	12	25.53
**Niveau socio-économique**		
Bons	557	46.07
Modeste	652	50.58

### Caractéristiques descriptives néonatales

L’étude des caractéristiques épidémiologiques des nouveaux nés décédés (Tableau 3) a révélé que 82.96% des nouveau-nés étaient de sexe masculin de faible poids de naissance (84.27%), plus particulièrement de très faible poids de naissance chez 456 cas. Pour la période de survenue des décès, 96,85% sont intervenues au cours de la période néonatale précoce comprise entre 0 et 7 jours inclus. En ce qui concerne l’âge gestationnel, 89% étaient des prématurés. 84.28% des nouveaux nées décédés avaient un score d'Apgar bas (<6).

**Tableau 3:** Description des caractéristiques néonatales des nouveau-nés décédés

Caractéristiques néonatales.	Nombre	Pourcentage%
**Poids**		
<1500g	456	37.71
1500-2500g	563	46.56
2500-4000g	126	10.42
>4000g	64	5.29
**Sexe**		
Masculin	1003	82.96
Féminin	199	16.45
Ambigüité sexuelle	7	0.57
**Score d'Apgar**		
0-5	1019	84.28
6-7	135	11.26
8-10	55	0.003
**Délai de survenue des décès**		
<24h	1008	83.37
1j-7 j	163	13.48
7 j-27 j	38	31.43
**Etiologie des décès**		
Age gestationnel <37SA	1076	94.96
DRS	15	1.32
INN	7	0.61
MMH	8	0.70
Asphyxie	10	0.88
Arrêt cardiaque	9	0.79
Malformation congénitale	8	0.70

### Facteurs de risque associés à la mortalité néonatale dans la région de Sidi Bel Abbes

Les variables associées à la mortalité néonatale au seuil p< 0,05 à l'analyse uni-variée ont été incluses dans un modèle de régression logistique pour déterminer les facteurs qui lui sont indépendants. Il en ressort de cette analyse que les facteurs de risque maternelle statistiquement associés au décès du nouveau-né (résume dans le n°04) était: l'Age de la mère >35 ans, la primiparité et la multiparité, l'HTA et l'infection génitale, les antécédents d'avortements, le niveau socio-économique modeste. Tandis que les facteurs de risques néonatales étaient majorée par: la prématurité, le faible poids de naissance, l'ictère et la souffrance f'tale aigue (Tableau 4).

**Tableau 4:** Facteurs de risque associés a la mortalité néonatale

Facteurs de risques	Nombre(%)	OR (IC 95%)	P
-Age de la mère			
**>35 ans**	644 (53.26)	3.1 (2.30 -4.40)	<0.001
-Parité			
**1**	323(26.71)	1.89 (1.01-2.53)	<0.001
**>3**	635 (52.52)	8.15 (2.85-10.05)	<0.001
-Pathologies associée A la grossesse			
**HTA**	44 (26.82)	2.6 (1.19-3.47)	<0.001
**INF**	81 (49.39)	5.3 (2.5-6.7)	<0.001
-Antécédent obstétricaux			
**ABRT**	20 (42.55)	2.03 (1.2-3.64)	<0.001
-Niveau socio-économique modeste	652(50.58)	1.5 (0.82-2.13)	<0.001
-Etiologie néonatale			
**Prématurité**	1076(89)	10.08 (3.45- 2.02)	<0.001
**FPN (<2500g)**	1019 (84.27)	4.5 (1.6-10.5)	<0.001
**Ictère**	387(32)	4.8 (1.26- 6.02)	<0.001
**SFA**	251(21)	3.4 (0.89- 5.14)	<0.001

## Discussion

Les décès néonatals représentent désormais environ 50% du total mondial des décès d'enfants de moins de cinq ans et plus de la moitié de la mortalité infantile. Le taux de la mortalité néonatale (MNN) au niveau du service de néonatologie de l'Etablissement Hospitalier Spécialisé Gynécologie Obstétrique (E.H.S.P.G) de la région de Sidi Bel Abbés durant la période d’étude allant de 2011-2012 était de 5.3%. Ce taux est relativement faible par rapport à 13% [5], 15% [6], 16,4% [7], et de 8,13% [8], à partir d’études menées, au Nigeria, la France, l'Inde et le Pakistan, respectivement. Une telle estimation peut être expliquée par le cadre de notre étude dans une région urbaine de l'Ouest algérien où par la prise en charge pluridisciplinaire au sein de l’établissement hospitalier de cette région. Sans oublier l'engagement effectif du gouvernement en faveur des soins de sante maternelle, infantile et néonatale. Pour les facteurs maternels, Portal B et al [9] trouvent qu’à un âge supérieur ou égal à 35 ans, le risque de mortalité fœtale et néonatale est multiplié par 3. En effet, l’âge maternel avancé a été démontré associé avec le pré éclampsie, d'autres maladies hypertensives, le diabète sucré, le faible poids de naissance et la prématurité [10, 11]. Dans notre étude, la moralité néonatale MNN intéressait 53.26% des cas de parturientes âgées >35 ans. De même, en France, Il a été rapporté que la mortalité néonatale était élevée chez les femmes âgées de moins de 20 ans et chez celles de plus 35 ans [12]. Dans la mortalité néonatale précoce, Senecal J et al. [13] trouvent une incidence plus élevée dès la 5eme grossesse. Portal B. et al [9] rapportaient également que la parité (superieur ou égale a 5) multiplie la mortalité par deux. Dans notre étude, la fréquence de la mortalité néonatale était importante chez les multipares soit à 52.52%. Ce qui corrobore avec les résultats soulignés au Mali et dont le taux le plus élevé de mortalité a été constaté chez les multipares soit à 60% [14]. La probabilité d'augmentation de la mortalité néonatale avec la parité pourrait être due au fait qu'avec l'agrandissement de la taille de la famille, les ressources des parents pourraient être insuffisantes pour maintenir un bon niveau de nutrition de plus d'enfants, et même la mère pourrait être en état sous-alimentés pendant sa grossesse. De même, le risque de complications obstétriques tend à augmenter avec la multiparité et ainsi augmente le risque de mortalité néonatale. Un faible intervalle inter génésique de moins de 24 mois a été également démonté associé à un risque accru de mortalité neonatale en raison du syndrome d’épuisement maternel [15, 16]. Malheureusement et vue le caractère rétrospective de notre étude nous n'avons pas pu étudier ce paramètre. Les complications sur grossesse ainsi que les antécédents maternelles influencent largement le pronostic néonatal. L'HTA constitue un facteur de risque majeur de mortalité maternelle et périnatale [17, 18]. Dans notre étude 26.82% soufraient d'HTA, ces résultats sont presque huit fois élèves par rapport à ceux noté en France soit à 2,4% [12]. Les infections maternelles augmentent le risque de mortalité périnatale. Dans notre série l'infection génitale (49.39%) était la première cause maternelle de mortalité néonatale. En effet, l'infection génitale peut augmenter le risque de rupture prématuré des membranes et donc de prématurité et de morbidité néonatale [19]. La mortalité périnatale est plus élevée chez les femmes ayant par exemple des antécédents d'avortement ou d'enfants décédés [20]. Dans notre série, 42.55% des parturientes présentaient un antécédent d'avortement alors que 25.53% avaient antécédents d'enfants décédés. Il faux noter que la mortalité néonatale varie en fonction de la classe sociale des parents comme l'ont rapportés certains auteurs [12]. En effet, le niveau socioéconomique bas, associé a la dénutrition maternelle, l'anémie et aux différentes complications obstétricales ont été démontré associé a un pronostic néonatale défavorable [10, 21]. Dans notre étude la majorité des parturientes provenaient d'un milieu socio-économique modeste.

Contrairement a la majorité des études qui considèrent la césarienne comme un facteur protecteur de décès néonatal et permettrait même d’éviter a 71% la mortalité périnatals [22]; dans notre étude ce mode d'accouchement a été pratique pour 65.83% des cas. Ce qui souligne le pronostic néonatal défavorable de nos parturientes, puisque dans ce service la césarienne n'est indiquée que dans le cas de grossesses a haut risque. La grande majorité des décès (96,85%) sont intervenue au cours de la période néonatale précoce comprise entre 0 et 7 jours inclus, ce qui corrobore les trouvailles de certains auteurs [23–25]. Une surmortalité néonatale masculine est constatée dans la plus part des donnes de la littérature [26]. Dans notre étude, 82.96% des nouveau-nés décédés étaient de sexe masculin contre que 16.45% de sexe féminin. Ce taux est plus élevé à celui noté au Cameroun à 70% [27]. On estime en général que le poids à la naissance est l'un des meilleurs indicateurs de chance de survie d'un nouveau-né. Les études ont montré qu'il existe une forte corrélation entre la mortalité néonatale et le faible poids à la naissance [28]. Dans notre étude les résultats montrent que le taux de la MNN liée au faible poids à la naissance était de 84.27% concordant avec ceux révélés par les études réalisées au République démocratique du Congo dont le taux était de 89% [29], au Camerone 80% [27], et en France 89.8% [12]. Plusieurs études ont montre une forte association entre la mortalité néonatale précoce et le score d'Apgar bas [30]. Dans notre étude, 84.28% des nouveau-nés décédés avaient un score d'Apgar bas (<6). En revanche, dans l'étude de Blondel et al. (2004) menée en France 75.6% avaient un scope d'apgar ≥ 7 [12].

Par ailleurs, la mortalité néonatale reconnue comme un fléau mondial. Les causes sont nombreuses et il peut y avoir une intrication de plusieurs facteurs. Les trois principales causes directes de mortalité néonatale en Afrique sont par ordre de grandeur, la prématurité, l'infection néonatale et l'asphyxie néonatale [31]. Dans notre série, les étiologies prédominantes étaient par ordre de fréquence les suivantes: la prématurité, le faible poids de naissance, l'ictère et la souffrance fœtale aigue. Dans son étude, Ngoc a indiqué que 42% des MNN était associé a la prématurité, 23% a l'asphyxie et 13% a des anomalies congénitales [32]. De sa part, Edmond [33, 34] a rapporté que l'asphyxie à la naissance était responsable de 42% des MNN, alors que la prématurité et les infections étaient chaque un responsable de 24% de MNN. Enfin, bien que les limites associées au caractère rétrospective de l’étude, dont le recueil des donnes n'a pas été exhaustif du fait des informations manquantes dans certains dossiers cliniques; cette étude nous a comme même permis de souligner les problèmes réels de morbidité et de mortalités posés a la sante du nouveau-né dans la région de Sidi Bel Abbes.

## Conclusion

La mortalité néonatale, bon indicateur de l’état de santé de l'enfant, constitue un véritable drame dans les populations les plus pauvres de chaque pays. L'Algérie comme tous les autres pays du tiers monde est confronté à ce drame silencieux qui représente un problème majeur de santé publique. Au terme de notre étude nous avons enregistré 1209 cas de mortalité néonatale durant les deux années (2011-2012), soit une fréquence de 5.3% et sont le résultat de la conjonction de différentes causes et facteurs de risque. Il s'agissait dans 96,85% des cas de mortalité précoce. Un renforcement des surveillances avant et pendant la grossesse, durant l'accouchement et même pendant la période du post partum; ainsi que une prise en charge efficace du nouveau-né dans sa première semaine de vie, devraient amélioraient le pronostic néonatal.

## References

[1] Zupan J (2005). Perinatal mortality in developing countries. N Engl J Med..

[2] Ngoc NT, Merialdi M, Abdel-Aleem H (2006). Causes of stillbirths and early neonatal deaths: data from 7993 pregnancies in six developing countries. Bulletin of the World Health Organization..

[3] OMS Le dossier mère enfant: Guide pour une maternité sans risque. http://WWW.Whaint/reproductive-health/publication/french-msm-94-11-msm-94-11.

[4] Berrah MK (2012). Dermographie Algérienne. Direction Technique chargée des statistiques de Population et de l'Emploi ONS_Avenue Belkacemi Mohamed_El Annasser Alger.

[5] Owa JA, Osinaike AI (1998). Neonatal morbidity and mortality in Nigeria. Indian J Pediatr..

[6] Kouéta F, Yé D, Dao L, Néboua D, Sawadogo A (2004). Neonatal morbidity and mortality in 2002-2006 at the Charles de gulle pediatric hospital (France). Child Care Health Dev..

[7] Oestergaard MZ, Inoue M, Yoshida S, Mahanani WR, Gore FM (2011). Neonatal Mortality Levels for 193 Countries in 2009 with Trends since 1990: A Systematic Analysis of Progress, Projections, and Priorities. PLoS Med..

[8] Nabeel M, Bushra M, Anum Y, Muneer A, Jai K (2012). The study of etiological and demographic characteristics of neonatal mortality and morbidity - a consecutive case series study from Pakistan. BMC Pediatrics..

[9] Portal B, Favard A, Suzanne F, Bandon J (1980). Etude de la mortalité fœtale per partum à la maternité de Clermont-Ferrand: A propos d'une série de 69 cas sur 5 ans (1973-1977). J Gynecol Obstet Biol Reprod..

[10] Seidman DS, Samueloff A, Mor-Yosef S, Schenker JG (1990). The effect of maternal age and socioeconomical background on neonatal outcome. Int J Gynaecol Obstet..

[11] Milner M, Barry-Kinsella C, Unwin A, Harrison RF (1992). The impact of maternal age on pregnancy and its outcome. Int J Gynaecol Obstet..

[12] Blondel Breart G (2004). Mortinatalité et mortalité néonatale: description facteurs de risque et évaluation des soins. EMC Obstétrique.

[13] Senecal J, Buestel ML, Delahaye M, Vongsavan Thong S, Lety A (1977). Etude de la mortalité néonatale précoce dans le département d'Ille-et- Vilaine en 1972-1973-1974. Ann Pediatr..

[14] Sidibé T, Sangho H, Doumbio S, Sylla M, Keita (2004). Mortalité néonatale dans discrict sanitaire de Kalokanie. Mali-Elsevier Masson. Journal de pédiatrie et de puériculture..

[15] Winkvist A, Rasmussen KM, Habicht JP (1992). A new definition of maternal depletion syndrome. Am J Public Health..

[16] Alger LS, Pupkin MJ (1986). Etiology of preterm premature rupture of the membranes. Clin Obstet Gynecol..

[17] Lansac J, Beger C, Magnin G (1990). HTA et grossesse. Obstétrique pour le praticien..

[18] Thiam M, Goumbala M, Gning SB, Fall P, Cellier C, Perret J-L (2003). Pronostic maternel et fœtale de l'association hypertension et grossesse en Afrique sub-saharienne (Sénégal). Journal de Gynécologie Obstétrique et Biologie de la Reproduction. Février.

[19] Kass E, Cormack WW, Lin J, Rosner B, Mynoz A (1981). A genital mycoplasmas as a cause of excess premature delivery. Trans Assoc Am Physicians..

[20] Rakotoseheno H, Rakotonirina EJ, Randriatsarafara FM, Rakotonantoanina J, Randrianarimanana VD, Rakotomanga JDM, Ranjalahy Rasolofomanana J (2008). Consultations prénatales et mortalité périnatale à Madagascar. Journal de Gynécologie Obstétrique et Biologie de la Reproduction..

[21] Jonas O, Roder D, Chan A (1992). The association of maternal and socio-economic characteristics in metropolitan Adelaide with medical, obstetric and labour complications and pregnancy outcomes. Aust NZ J Obstet Gynaecol..

[22] Labie D (2005). Le scandale des 4 millions de morts néonatales chaque année: Bilan et actions possibles. Médecine/sciences..

[23] Lawn JE, Cousens S, Zupan J (2005). Lancet Neonatal Survival Steering Team. 4 million neonatal deaths: When? Where? Why?. Lancet.

[24] Chowdhury HR, Thompson S, Ali M (2010). Causes of Neonatal Deaths in a Rural Sub district of Bangladesh: Implications for Intervention. J Health Popul Nutr..

[25] David C, Francisca M, Francine Romyale TN, Félix T (2012). Mortalité néonatale précoce et ses déterminants dans une maternité de niveau I à Yaoundé, Cameroun. Pan African Medical Journal..

[26] Akaffou E, Amon tanoh-dick F, Lasme-guillao BE, Yenan JP (2011). Mortalité néonatale et niveaux de diagnostic au centre hospitalier universitaire de Yopougon (Abidjan). Mali médical..

[27] Kedy D, Koum, Exhenry C, Penda CI, Nzimanzima, Pfister RE (2012). Morbidité et mortalité néonatal dans un hospital de discrict urbain à resources limitées à Douala Cameroun. EMC.

[28] Koko J, Dufillotd D, Ganhouma A (2002). Moussavou, Facteurs de mortalité des prématurés dans le service de pédiatrie de l'hopital péditrique d'Owendo-Libreville(Gabon). Archives de pédiatrie..

[29] Ntambue A, Malonga F, Dramaix M, Donnen P (2013). La mortalité périnatale: ampleur et causes à Lubumbashi République Démocratique du Congo-Revu d’épidémiologie et de santé publique. Elsevier Masson-Télécharger 30-03-2014 par SCD université d'Angers.

[30] Alihonou E, Dan V, Ayivi B, Sossou E, Gandaho T, koumakpai S (1991). Mortalité néonatale au centre national hospitalier et universitaire de Cotonou: incidence, causes et moyens de lutte. Médecine d'Afrique noire..

[31] World Health Organization (2006). Neonatal and Perinatal Mortality Country, Regional and Global Estimates.

[32] Ngoc NT, Merialdi M, Abdel-Aleem H (2006). Causes of stillbirths and early neonatal deaths: data from 7993 pregnancies in six developing countries. Bulletin of the World Health Organization..

[33] Edmond K, Quigley M, Zandoh C (2008). Aetiology of stillbirths and early neonatal deaths in rural Ghana: implications for health programming in developing countries. Paediatric and Perinatal Epidemiology..

[34] Edmond K, Quigley M, Zandoh C (2008). Diagnostic accuracy of verbal autopsies in ascertaining the causes of stillbirths and neonatal deaths in rural Ghana. Paediatric and Perinatal Epidemiology..

